# First Anti-Inflammatory Peptide AnmTX Sco 9a-1 from the Swimming Sea Anemone *Stomphia coccinea*

**DOI:** 10.3390/biom12111705

**Published:** 2022-11-17

**Authors:** Rimma S. Kalina, Irina N. Gladkikh, Anna A. Klimovich, Yulia V. Kozhevnikova, Aleksandra N. Kvetkina, Eugene A. Rogozhin, Sergey G. Koshelev, Sergey A. Kozlov, Elena V. Leychenko

**Affiliations:** 1 G.B. Elyakov Pacific Institute of Bioorganic Chemistry, Far-Eastern Branch of the Russian Academy of Science, 159, Pr. 100 let Vladivostoku, Vladivostok 690022, Russia; 2 Shemyakin-Ovchinnikov Institute of Bioorganic Chemistry, Russian Academy of Sciences, ul. Miklukho-Maklaya 16/10, Moscow 117997, Russia

**Keywords:** sea anemone, beta-hairpin peptides, Edman sequencing, analgesic and anti-inflammatory activity, TNF-α

## Abstract

A novel peptide AnmTX Sco 9a-1 with the β-hairpin fold was isolated from the swimming sea anemone *Stomphia coccinea* (Actinostolidae family). The peptide consists of 28 amino acid residues, including modified hydroxyproline residue, and its measured molecular mass is 2960 Da. The peptide was not toxic on mice; however, it stimulated their exploratory motivation and active search behavior, and demonstrated an anti-anxiety effect. AnmTX Sco 9a-1 at doses of 0.1 and 1 mg/kg reduced the volume of edema during 24 h better than the nonsteroidal anti-inflammatory drug, Diclofenac, at dose of 1 mg/kg in a model of acute local λ-carrageenan-induced inflammation. ELISA analysis of the animal’s blood showed that peptide at a dose of 1 mg/kg reduced the content of tumor necrosis factor-α (TNF-α), a pro-inflammatory mediator responsible in the edema development, up to the level of TNF-α in the intact group. Besides, AnmTX Sco 9a-1 demonstrated a significant analgesic effect on acute pain sensitivity in the carrageenan-induced thermal hyperalgesia model at doses of 0.1 and 1 mg/kg. Activity of AnmTX Sco 9a-1 was shown not to be associated with modulation of nociceptive ASIC channels.

## 1. Introduction

Inflammation is an essential protective response of the organism to a mechanical, thermal, and chemical stimulus or to pathogenic agents. If the acute phase of inflammation fails to remove the inflammatory stimulus (infectious agent or foreign material) or injury-damaged cells and initiate healing, the inflammation becomes persistent and then progresses to the chronic phase [1]. Since chronic inflammation has been reported to contribute to the onset and development of numerous diseases, including arthritis, asthma, atherosclerosis, autoimmune diseases, diabetes, cancer, and neurodegenerative diseases [2], early anti-inflammatory therapy can prevent the development of chronic pain.

Inflammation is a complex reaction accompanied by undesirable manifestations like redness, heat, edema, and pain. During inflammation, damaged tissues release inflammatory mediators such as bradykinin, serotonin, histamine, nerve growth factor, prostaglandins, neuropeptides, and cytokines, as well as adenosine triphosphate (ATP) and reactive oxygen species (ROS) to activate both immune and neuronal cells [3]. In neurons, the inflammatory mediators can affect the expression and function of nociceptive ion channels, such as sodium, chloride, calcium, potassium, transient receptor potential and purinergic and acid-sensitive ion channels followed by a complete change in a total excitability of peripheral nociceptive fibers, which leads to inflammatory pain [4,5]. In this regard, the search of new effective ion channels modulators can help in the development of anti-inflammatory drugs.

One promising source of biologically active compounds is a venom of marine intestinal animals—sea anemones (Cnidaria, Anthozoa, and Actiniaria), which produce a large number of active peptides (toxins) affecting different cellular targets and described in the following reviews [6,7,8,9,10,11]. The main biological functions of such toxins are defense against natural predators, species competition, and weapons for hunting [7].

Today many molecules without toxicity to mammalians were purified from Actiniaria that are able to reduce inflammation and hyperalgesia: for example, Kunitz-type peptides from the sea anemone *Heteractis magnifica* (=*Heteractis crispa*), which can decrease histamine- and carrageenan-induced inflammation in in vivo models [12,13,14] and reduce the content of ROS or cytokines in macrophages treated by lipopolysaccharide [15,16,17] and neuronal cells treated by neurotoxins [14,18,19]. In addition, Kunitz-type peptides are able to suppress thermal and mechanical hypersensitivity in the in vivo models of carrageenan-induced inflammation [14,16], monosodium iodoacetate-induced osteoarthritis and Complete Freund’s Adjuvant (CFA)-induced rheumatoid monoarthritis [20].

Sea anemone *Stomphia coccinea*, a largely unexplored species without active peptides isolated is the object of current research. There is only one publication describing identification of actinoporin-like genes in five species of sea anemones including *S. coccinea* using a combined bioinformatic and phylogenetic approach [21]. Here, we report a novel peptide of *S. coccinea*, AnmTX Sco 9a-1, shared a so-called uncommon β-hairpin fold [22], expressing anti-inflammatory and analgesic effects in a mouse model of carrageenan-induced paw edema. Additionally, structurally related peptides isolated from sea anemones are known as analgesic molecules; these are the toxin AnmTX Ms 9a-1 and AnmTX Ugr 9a-1. Ms 9a-1 is a positive modulator of the transient receptor potential ankyrin 1 (TRPA1) channels. It decreases the nociceptive and inflammatory response to allyl isothiocyanate (the agonist of TRPA1) and reverses CFA-induced inflammation and thermal hyperalgesia [23]. Ugr 9a-1 is an inhibitor of the acid-sensing ion channels (ASICs), and is known to reverse acid- and CFA-induced inflammatory pain [22]. A novel peptide AnmTX Sco 9a-1 is set apart from its known homologs by the following characteristics: more than 50% of sequence difference, the molecular mechanism of anti-inflammatory and antinociceptive action (different from those of Ugr 9a-1; the involvement of TRP receptors is currently unknown), and an anti-anxiety effect, which was a first-time find for the sea anemones’ β-hairpin toxins.

## 2. Materials and Methods

The sea anemones *S. coccinea* (Müller, 1776) (order Actiniaria, family Actinostolidae) were collected in the Sea of Okhotsk on the insular shelf and slope of the Iturup Island during cruises of the R/V Akademik Oparin no. 47 (2015). The species of sea anemone was identified morphologically by Dr. E. Kostina (A.V. Zhirmunsky National Scientific Center of Marine Biology FEB RAS) as well as phylogenetically on the basis of nuclear and mitochondrial markers [24]. Sea anemones were frozen and kept at −20 °C.

### 2.1. Isolation of the Sea Anemone Peptide

Peptides were extracted from whole bodies of sea anemone *S. coccinea* with 96% ethanol for 24 h. The extract was filtered and centrifuged for 15 min at 10,000 rpm (Eppendorf, Hamburg, Germany). After ethanol evaporation, the supernatant was lyophilized and stored at –20 °C. At first, hydrophobic chromatography on a polychrome-1 (powdered Teflon, Biolar, Olaine, Latvia) column (4.5 cm × 7 cm) was performed using elution with water and 40% aqueous ethanol at 5 mL/min flow rate and 5 mL fractions were collected at 6 °C. The absorbance of eluent was monitored at 280 nm. Further hydrophobic peptides eluted by 40% ethanol were separated on reversed-phase Luna C18 column (10 mm × 250 mm) (Phenomenex, Torrance, CA, USA) equilibrated with 10% acetonitrile (Cryochrom, St-Petersburg, Russia) solution with 0.1% trifluoroacetic acid (TFA, PanReac AppliChem, Barcelona, Spain) on an Agilent 1100 chromatograph (Agilent Technologies, Santa Clara, CA, USA) at 1.5 mL/min flow rate. Peptides elution was carried out using combined gradient of acetonitrile concentration from 10 to 40% for 30 min, then 40% of acetonitrile. The absorbance of eluent was monitored at 214 nm. The final separation of the peptide was done on the C18-PFP column (4.6 mm × 250 mm) (Advanced Chromatography Technologies Ltd., Aberdeen, United Kingdom) equilibrated with 10% acetonitrile solution with 0.1% TFA according to the scheme: from 10 to 25% of acetonitrile for 15 min, then 25% of acetonitrile at 1 mL/min flow rate. The vacuum concentrator 5301 (Eppendorf, Hamburg, Germany) was used for acetonitrile evaporation.

### 2.2. Mass Spectrometric Analysis

A mass spectrometric analysis was carried out using an Ultraflex TOF/TOF mass spectrometer (Bruker Daltonik, Karlsruhe, Germany) and a quadrupole time-of-flight mass spectrometer MaXis impact (Bruker Daltonik, Karlsruhe, Germany) equipped with MALDI and ESI ionization sources, respectively. The sample was solved in an acetonitrile/water solution (1:1, *v*/*v*) containing 0.1% TFA. For MALDI analysis, 10 mg/mL of sinapinic acid as a matrix was used.

### 2.3. Reduction and Alkylation of Disulfide Bridges

Peptides were reduced and alkylated with 4-vinylpyridine (Sigma Aldrich, St. Louis, MO, USA) as described in [25]. The reaction mixture was separated on a reversed-phase Nucleosil C18 column (4.6 mm × 250 mm) (Sigma Aldrich, St. Louis, MO, USA) equilibrated with 10% acetonitrile with 0.1% TFA. The elution was carried out using a gradient of acetonitrile concentration (10% of acetonitrile for 30 min; from 10 to 40% for 60 min) at 0.5 mL/min flow rate.

### 2.4. Sequence Determination and Primary Structure Analysis

The primary structure of the modified peptide (approximately 800 pmoles) was determined using an automated Edman degradation on a PPSQ-33A protein sequencer (Shimadzu Corp., Kyoto, Japan) according to the manufacturer’s protocol. Amino acid residues were identified as their phenylthiohydantion (PTH) derivatives by analytical reversed-phase HPLC compared with retention times of standard PTH-amino acids (Wako Pure Chemicals GmbH, Neuss, Germany). Three hundred pmoles of L-hydroxyproline analytical grade (Serva, Heidelberg, Germany) were used as the standard. Data analysis was carried out using the LabSolutions software version 1.10 (Shimadzu Corp., Kyoto, Japan). The sequences’ similarity was analyzed using amino acid sequence databases and the BLAST algorithm [26]. Multiple alignment of amino acid sequences was made using Vector NTI software (Invitrogen, Eugene, OR, USA).

### 2.5. Hemolytic Activity Assay

Hemolytic activity was detected in a 0.7% solution of mouse erythrocytes in a medium containing 0.9% NaCl, 1 mM KCl, and 10 mM glucose. Then, 0.01 mL of targeting fraction was mixed with 0.09 mL of erythrocyte suspension, and the mixture was incubated for 1 h at 37 °C. Hemoglobin level in the supernatant was spectrophotometrically measured at λ = 540 nm after the preliminary rapid cooling of the reaction mixture and its centrifugation to precipitate erythrocytes and their shadows. The optical density of the supernatant (0.8) of the control specimens, where the lysis of erythrocytes was induced by the addition of 0.01 mL 1% solution of holothurin A1 from sea cucumber *Eupentacta fraudatrix* [27], was assumed to be 100% hemolysis.

### 2.6. Electrophysiology

Rat ASIC1a and ASIC3 channels were expressed in *X. laevis* oocytes after injection of 2.5–10 ng of cRNA, as previously described [28]. After injection, oocytes were kept for 2–3 days at 19 °C and then up to 5 days at the temperature of 15–16 °C in sterile ND96 medium (96 mM NaCl, 2 mM KCl, 1.8 mM CaCl_2_, 1 mM MgCl_2_ and 5 mM HEPES, titrated to pH 7.4 with NaOH supplemented with 50 μg/mL of gentamycin). Two-electrode voltage clamp recordings were performed using a GeneClamp 500 amplifier (Axon Instruments, Burlingame, CA, USA). The data were filtered at 50 Hz and digitized at 1000 Hz with an AD converter L780 (LCard, Moscow, Russia) using in-house software. The solutions were applied to a cell chamber (volume 50 μL). The laminar flow of an external solution of ND96 (pH 7.4) was used at a rate of 1 mL/min. ASIC1a and ASIC3 were activated with a short (1 s) application of a solution with a pH of 5.5 (10 mM MES) using a fast application system. Peptides were applied 15 s before the activation pulse in a solution containing 0.1% BSA.

### 2.7. Animal Studies

The animal studies were performed under the European Commission’s legislation for the protection of animals used for scientific purposes (Directives 86/609/EEC, 2010/63/EU), the National Standard of the Russian Federation “Good Laboratory Practice” (GOST P 53434-2009, Moscow, Russia), and was approved by G.B. Elyakov Pacific Institute of Bioorganic Chemistry (Far Eastern Branch, Russian Academy of Sciences) Committee on Ethics of Laboratory Animal Handling 01/22, 4 August 2022 protocol. Adult female CD-1 line white mice weighing 25 ± 2 g were kept at room temperature with a 12 h light/dark cycle and with ad libitum access to food and water. There were seven or eight individuals in each group. In total, 133 mice were used for the study.

#### 2.7.1. Acute Toxicity

Lyophilized peptide fraction was dissolved in 0.9% sterile NaCl solution and administered once intravenously at doses of 15 mg/kg; the control group received saline (0.9% NaCl, 10 mL/kg or 0.250 mL/mouse). Then, changes in basic physiological parameters, such as motility, behavioral responses, and physical activity, were registered in each group of animals over the course of 24 h.

#### 2.7.2. Tail-Flick Test

The analgesic activity of peptides in the fraction was assessed in a tail-flick test. Lyophilized peptide fraction was dissolved in 0.9% sterile NaCl solution (1 mg/mL) and administered intramuscularly into the root of the tail, 100 µL/mouse. Control animals received 0.9% sterile NaCl solution 100 μL/mouse. For the tail-flick test, one hour after injection the mouse was restrained in a soft tissue pocket, and the distal half of the tail was immersed into water heated up to 50 °C. The pain threshold was detected as latency time to the tail withdrawal. Latency for tail-flick was measured with a 10-s cutoff time to avoid animal injury.

#### 2.7.3. Open Field Test

Neurotropic, irritating or depressive effects of the peptide on the central nervous system, and effects on the motor and orienting-exploratory activity of animals were assessed at the Open Field facility (OpenScience, Krasnogorsk, Russia). The installation is a round gray PVC arena with a diameter of 63 cm, with a wall height of 32 cm. At the bottom of the arena there are 12 holes with a diameter of 1 cm, allowing researchers to explore the mink activity of rodents. The peptide was administered to animals intramuscularly (into the quadriceps muscle of the left thigh) at doses of 0.01, 0.1, and 1 mg/kg 60 min before testing. The control group was injected with an equivalent volume (0.05 mL) of sterile saline. 60 min after the injection, the animals were placed in the center of the illuminated arena and the recording of the behavior and movement of the animal on video was immediately started; the duration of the test was 3 min. Registration and analysis of video files to assess the behavior, movement and actions of rodents were carried out using a camera with the software “Minotaur”, (LLC “Neurobiotics”, Zelenograd, Russia). During testing, next parameters were recorded: T (act)—activity time; T (pass)—passivity time; T (c.z.)—time spent on the central zone, s; T (b.z.)—stay on the border zone, s; V, m/s—average travel speed; V(act), m/s—average movement speed during activity; S, m—distance traveled; S (act), m—distance traveled during activity; N (c.z.)—number of visits of central zone; N (s.p.)—number of visits of side platform; N (racks)—vertical activity, the number of racks; N (peeps)—holes explored, the number of peeps into minks; N (def)—number of bowel movements; T (e.c.z.)—time of exit from the central zone, s.

#### 2.7.4. Carrageenan-Induced Paw Edema

The anti-inflammatory activity of the peptides was assessed in a model of the carrageenan-induced paw edema. A peptide sample was dissolved in sterile saline and administered intramuscularly at doses of 0.01, 0.1, and 1 mg/kg 60 min before induction of inflammation. Control animals received an equivalent volume of sterile saline. Diclofenac (Hemofarm A.D., Vršac, Serbia), a commercial drug of the NSAID group, intended for the treatment of pain of various origins, including those caused by inflammatory processes at a dose of 1 mg/kg, was used as a positive control. Each mouse received 20 µL of a 1.5% solution of λ-carrageenan in the hind paw pad after 60 min. Then, the resulting edema was measured at several time points (1, 2, 4, and 24 h) using a plethysmometer (Ugo Basile, Gemonio (VA), Italy). The volume of the resulting inflammatory paw edema was calculated using the following formula:

Volume Growth Index (%) = [(Vc − Vi)/Vi] × 100,(1)

where Vc is the volume of the paw after the introduction of carrageenan, Vi is the volume of the paw before the introduction of carrageenan. The anti-inflammatory threshold was detected as a decrease of paw volume and Volume Growth Index (%) throughout the entire observation period.

#### 2.7.5. Animal Euthanasia Procedure and Blood Sampling

Animals were terminally anaesthetized with sodium pentobarbital (40 mg per mouse i.p., Euthatal, Merial Animal Health, Essex, UK) 24 h after carrageenan injection. Then, the thoracic cavity was opened and blood was collected in tubes with the ethylenediaminetetraacetic acid (EDTA, Sigma Aldrich, St. Louis, MO, USA) directly from the right atrium of the heart. The whole blood was clotted for two hours at room temperature and then was centrifuged at 1.0 × 10^3^× *g* for 20 min to remove cells; the blood serum was then aliquoted and stored at −20 °C.

#### 2.7.6. ELISA

The blood serum samples were analyzed for TNF-α in the enzyme-linked immunosorbent assay (ELISA) using a diagnostic kit according to the manufacturer’s protocol (Cloud-Clone Corp., CCC, Wuhan, China).

Blood serum samples and standard solution (100 µL) were added to the 96-well microplate pre-coated with anti-mouse TNF-α antibody and incubated for 1 h at 37 °C. After incubation the liquid was removed, 100 µL of a biotin-conjugated anti-TNF-α antibodies were added and incubated for 1 h at 37 °C. After incubation wells were washed for 3 times with 300 µL 1% wash buffer and all liquid was removed. Then, 100 µL of streptavidin-HRP solution was added and incubated for 30 min at 37 °C. After incubation the wash was repeated 5 times as previously described, and 100 µL of TMB substrate (tetramethylbenzidine with H_2_O_2_) was added and incubated for 10 min in the dark at room temperature until the emergence of blue color. Then, the 50 µL of stop solution (0.5 M H_2_SO_4_) was added and the absorbance at 450 nm was measured with the microplate reader BioMark xMark (Bio-Rad Laboratories, Inc., Hercules, CA, USA). Calculation of the TNF-α level was carried out using the calibration curve that was plotted as the absorbance of each standard solution (Y) vs. the respective concentration of the standard solution (X).

#### 2.7.7. Thermal Hyperalgesia

Thermal hyperalgesia was tested with a Hot-Plate Analgesia Meter (IITC Life Science Inc., Woodland Hills, CA, USA) set at 52 °C. The animals were placed individually on the preheated hot-plate surface and exposed to heat until nociceptive reaction was registered. The pain threshold was detected as latency to hind paw withdrawal or licking. The maximum residence time of the animal on the plate did not exceed 30 s. Measurements were carried out 1 h after the injection of λ-carrageenan.

#### 2.7.8. Statistic Calculation

All data are expressed as mean ± S.D. Student’s *t*-test was performed to determine statistical significance.

## 3. Results and Discussion

### 3.1. Peptides Isolation

**Figure 1:**
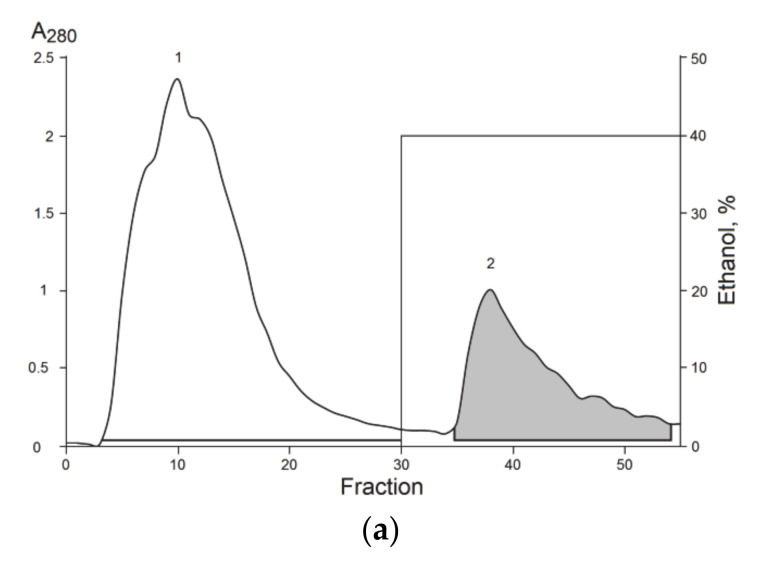

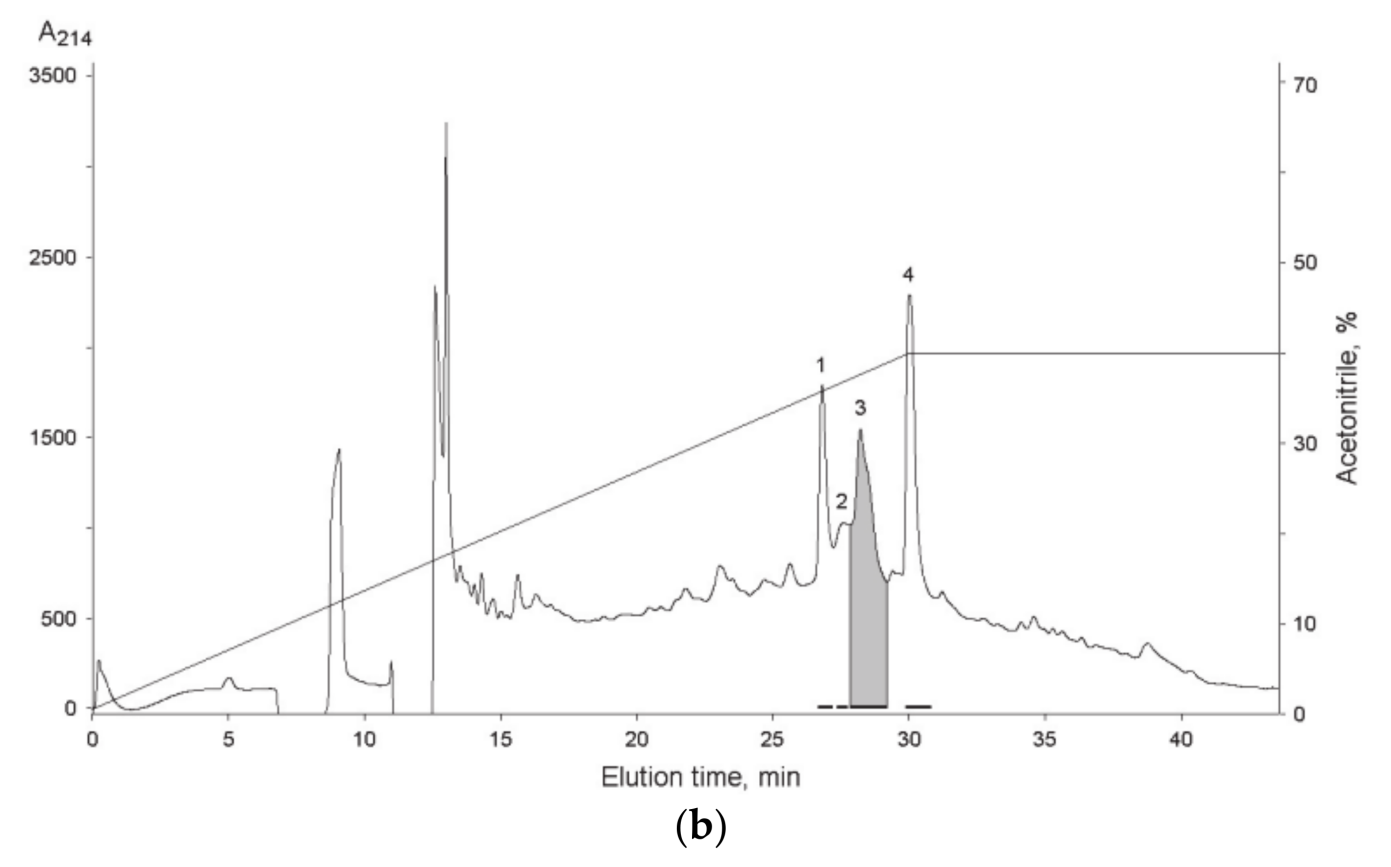
Isolation of *S. coccinea* bioactive compounds. (**a**) Hydrophobic chromatography of ethanol extract on polychrome-1 column (4.5 cm × 7 cm). 1—Hydrophilic fraction eluted with water; 2—hydrophobic fraction eluted with 40% aqueous ethanol. (**b**) RP-HPLC of fraction 2, obtained after the hydrophobic chromatography, on a Luna C_18_ (10 mm × 250 mm) column in a gradient of acetonitrile with 0.1% TFA. The numbers and solid lines under the peaks indicate collected fractions. Fractions from which the peptide was isolated are gray colored.

The chromatographic separation of molecules from the ethanol extract of *S. coccinea* was carried out in three stages. Supernatant after extraction (see material and methods for details) was separated at more hydrophilic compounds eluted from polychrome-1 column by water (Figure 1a, fraction 1) and hydrophobic ones eluted by 40% aqueous ethanol (Figure 1a, fraction 2). After the intramuscular injection of hydrophobic fraction 2 (4 mg/kg) in the root of mice tail we detected a considerably increase of the tail-flick latency when compared with the saline-treated control (data not shown). The basal reaction time of animals was in 2–4-s range. The stable analgesic effect developed within 1 h after administration. No toxicity to animals was indicated when the fraction 2 was injected intravenously at a dose of 15 mg/kg. So at least one active peptide non-toxic to mammalians should be presented in the extract obtained at first chromatographic stage.

The hydrophilic fraction 1 contained compounds with a hemolytic activity and was rejected for subsequent experiments.

**Figure 2:**
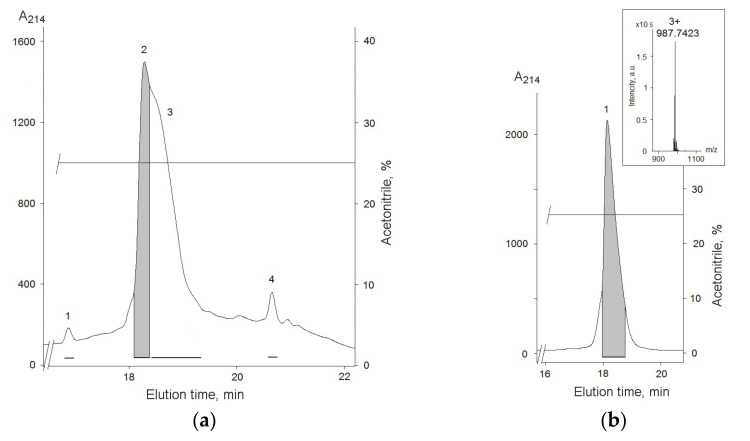
(**a**) RP-HPLC of fraction 3, obtained after the first step RP-HPLC, on a C18-PFP (10 mm × 250 mm) column in a gradient of acetonitrile with 0.1% TFA. (**b**) Purity check on RP-HPLC C18-PFP (10 mm × 250 mm) column. ESI MS measure of average molecular weight of peptide is shown in insert. Fractions from which the peptide was isolated are gray colored.

To isolate compounds with the analgesic activity second stage on RP-HPLC was used and the main four fractions were obtained (Figure 1b). According to MALDI-MS analysis fractions 1, 2, and 4 were mixtures of peptides with a molecular weight in 1.3–11.5 kDa range. On the contrary, fraction 3 (Figure 1b) contained mainly peptides with a molecular weight of about 2930–3010 Da. The major compound was finally purified from fraction 3 (Figure 2a) on the C18-PFP column and its average molecular weight, 2960.20 Da, was measured by ESI MS (Figure 2b). The 350 μg of peptide was obtained as the result of separation of 4.5 g of lyophilized ethanol extract.

### 3.2. Peptide Sequence Determination

**Figure 3:**
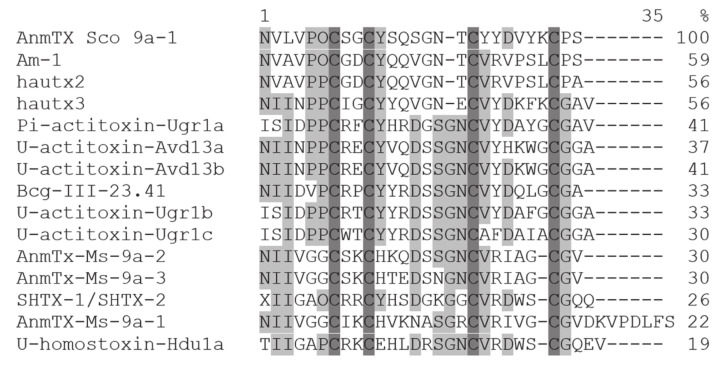
Multiple sequence alignment of the structural group 9a toxins: AnmTX Sco 9a-1 (C0HM64) *S. coccinea*; Am-1 (P69929) from *Antheopsis maculata* [31]; hautx2 (A0A0P0UTI6) and hautx3 (A0A0P0UTQ7) from *Heteractis aurora* [32]; Pi-actitoxin-Ugr1a, U-actitoxin-Ugr1b, and U-actitoxin-Ugr1c (R4ZCU1) from *Urticina grebelnyi* [22]; U-actitoxin-Avd13a and U-actitoxin-Avd13b (P0DMZ8) from *Anemonia viridis* [33]; Bcg-III-23.41 (P86467) from *Bunodosoma cangicum* [34]; AnmTx-Ms-9a-1, AnmTx-Ms-9a-2 (A0A1R3S3A8), and AnmTx-Ms-9a-3 from *Metridium senile* [23]; SHTX-1/SHTX-2 (P0C7W7) from *Stichodactyla haddoni* [35]; and U-homostoxin-Hdu1a (C0HJB4) from *Stichodactyla duerdeni* [36]. Modified residue–hydroxyproline shown as O in sequences. Identical and conserved amino acid residues are shown on a dark and light gray background, respectively. Vector NTI Advance v. 11.0 (Invitrogen, Carlsbad, CA, USA) [37] was used for multiple sequence alignment.

It is common for sea anemone toxins to have a structure stabilized by disulfide bonds. So a reduction of S-S bonds by dithiothreitol following alkylation with 4-vinylpyridine was performed. The modified peptide had molecular weight growth on 424 Da; it confirmed that there are two disulfide bonds in the native molecule. Complete amino acid sequence of the peptide was determined by an automatic Edman degradation. The peptide consists of 28 amino acid residues, and has modified residue – hydroxyproline (O) in position 6 (Figure 3 and Figure S1). This approach to determine hydroxyproline residue by Edman sequencing was previously applied to plant antimicrobial peptides [29]. The measured molecular weight of the peptide accurately correlated with theoretical one calculated from the sequence.

The swimming sea anemone *S. coccinea* is a largely unexplored species; there are several sequences similar to its toxins in the databases, but they all belong to the group of actinoporins [21]. According to the hemolytic activity of fraction 1 from polychrome-1 (Figure 1a), actinoporins were contained in this fraction. The highest homology of the peptide was observed with peptides from other structural group sharing a spatial fold stabilized by two disulphide bridges, with three classical beta turns and twisted β-hairpin unconnected by disulfide bridges, and called β-hairpin [22]. Therefore, the new peptide was named AnmTX Sco 9a-1 according the convenient nomenclature [30]. Such peptides in the sea anemone genome have been established to be encoded by a precursor protein containing several homologous peptides [23,30]. According to MS data, fraction 3 (Figure 2a) may contain such homologous peptides. However, apart from the major peptide AnmTX Sco 9a-1, it has not yet been possible to isolate any other individual peptide, but we believe to find them during further genomic/transcriptomic studies of this species.

According to the BLAST result, AnmTX Sco 9a-1 was similar by sequence to 14 peptides from eight sea anemone species of three families: *Urticina grebelnyi*, *Antheopsis maculata*, *Anemonia viridis*, and *Bunodosoma cangicum* (Actiniidae); *Heteractis aurora*, *Stichodactyla haddoni*, and *Stichodactyla duerdeni* (Stichodactylidae); and *Metridium senile* (Metridiidae) (Figure 3), while *S. coccinea* itself belongs to the Actinostolidae family. 

The sequence identity varied from 19% to U-homostoxin-Hdu1a from *S. duerdeni* up to 59% to Am-1 toxin from *A. maculata*. As well, post-translational modification in position 6 for AnmTX Sco 9a-1 was not exclusive and hydroxyproline residue was found in a similar position for peptides Am-1 and SHTX-1/SHTX-2 (Figure 3).

Cellular targets have been identified only for several homologous peptides of AnmTX Sco 9a-1. The Na_V_ channels are possible cellular targets of Am-1, since it was weakly lethal to crabs with LD_50_ values 830 mg/kg [31]. K_V_ channels were proposed to be affected by Bcg-III-23.41, which increases amplitude and duration of crab leg nerve compound action potential [34] and by SHTX-1/SHTX-2, which inhibits the binding of ^125^I-alpha-dendrotoxin to synaptosomal membranes (IC_50_ = 270 nM) [35]. Two other homologous peptides, AnmTX Ms 9a-1 and AnmTX Ugr 9a-1, producing analgesic and anti-inflammatory effects in in vivo models, are a positive modulator of the TRPA1 and an inhibitor of the ASIC3 channels, respectively [23,38].

### 3.3. Animal Expepriments

#### 3.3.1. Anti-Inflammatory Activity of AnmTX Sco 9a-1

The anti-inflammatory activity of the peptide was studied using a model of acute local inflammation induced by carrageenan which causes extravasation of neutrophils, synthesis of pro-inflammatory cytokines, oxidative stress, and activation of apoptosis of various epithelial cells, and leads to the formation of edema [39]. This method is widely used to evaluate the anti-inflammatory effect of potential immunomodulatory and analgesic drugs.

**Figure 4:**
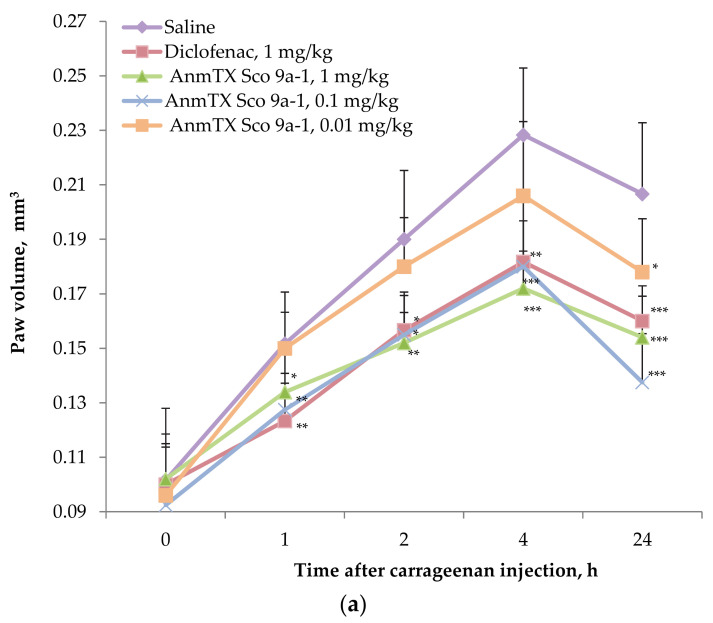

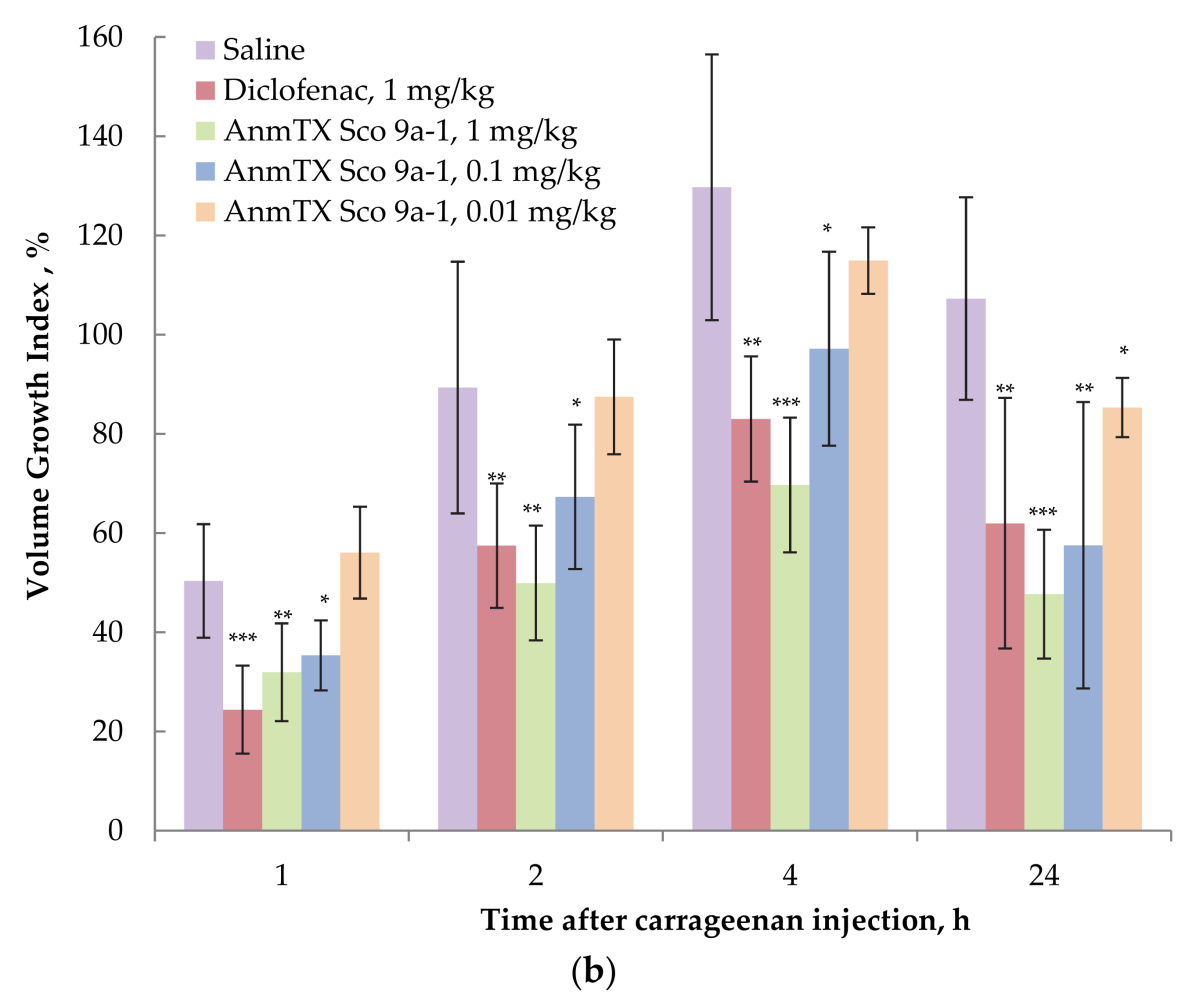
Time dependent effect of AnmTX Sco 9a-1 on (**a**) paw volume and (**b**) Volume Growth Index (%) in the carrageenan-induced inflammation model. A saline buffer as a negative control and Diclofenac at a dose of 1 mg/kg administered intramuscularly as a positive control were used. Results are presented as mean ± SD (n = 7–8). The significance was calculated by the Student’s *t*-criterion to the saline group and was marked as * *p* < 0.05, ** *p* < 0.01, and *** *p* < 0.001.

The peptide demonstrated a stable anti-inflammatory effect at doses of 1 and 0.1 mg/kg manifested in the paw volume decrease throughout the entire observation period (Figure 4a). At the same time, at a dose of 1 mg/kg, the peptide was superior by efficiency to the commercial drug Diclofenac at the same dose at longer measure points. As shown on Figure 4b the growth rate of inflamed paw volume at 1 h was decreased by Diclofenac more effectively, but the peptide at the dose of 1 mg/kg caught up to Diclofenac’s efficacy by 2 h and then was more effective at 4 and 24 h, demonstrating the volume growth reduction in two times. In animals treated with the peptide at dose of 0.01 mg/kg, a significant decrease in the paw volume and growth index was registered only 24 h after the induction of inflammation. Therefore, optimal dose of the peptides as anti-inflammatory agent was determined as 0.1 mg/kg for mice.

**Figure 5:**
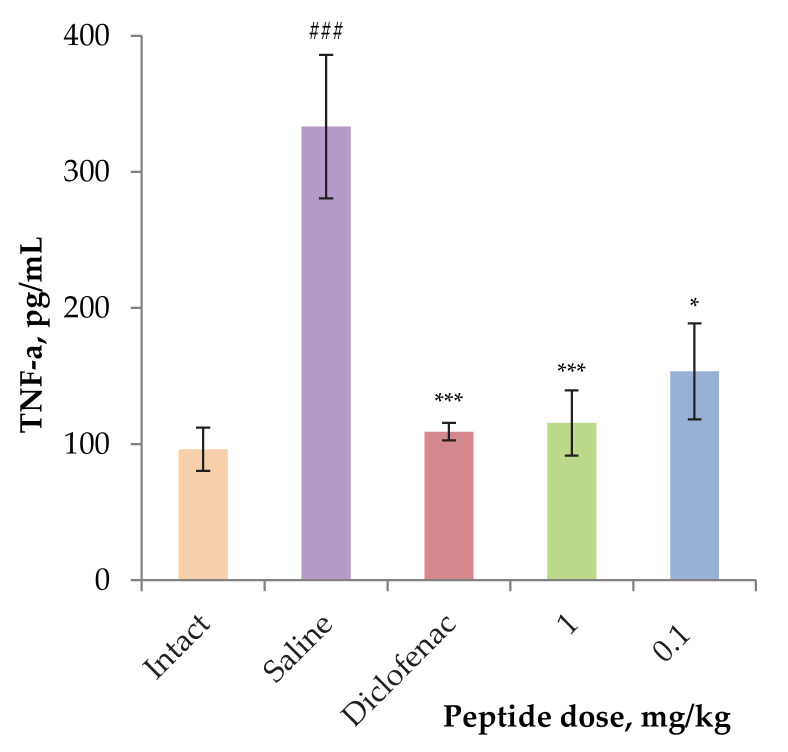
The level of TNF-α in the blood serum of experimental animals. Blood samples were obtained 24 h after induction of inflammation by carrageenan. Results are presented as mean ± SD (n = 7–8). The significance of differences was estimated by the Student’s *t*-criterion to the saline group (*) and to the intact serum (#). Significant differences are presented as * *p* < 0.05, *** *p* < 0.001, and ### *p* ≤ 0.001.

In order to assess the effect of the peptide on the level of inflammatory cytokines, in particular TNF-α, blood was taken from the mice of the groups treated with AnmTX Sco 9a-1 at doses of 0.1 and 1 mg/kg after 24 h. In response to the carrageenan administration, the TNF-α production in control group animals increased dramatically to more than three times the level in the intact group of animals. At the same time, animals treated by 1 mg/kg dose of Diclofenac or AnmTX Sco 9a-1 retained TNF-α level in blood same to intact animals (Figure 5).

#### 3.3.2. Effect of Peptide on Thermal Hyperalgesia

**Figure 6:**
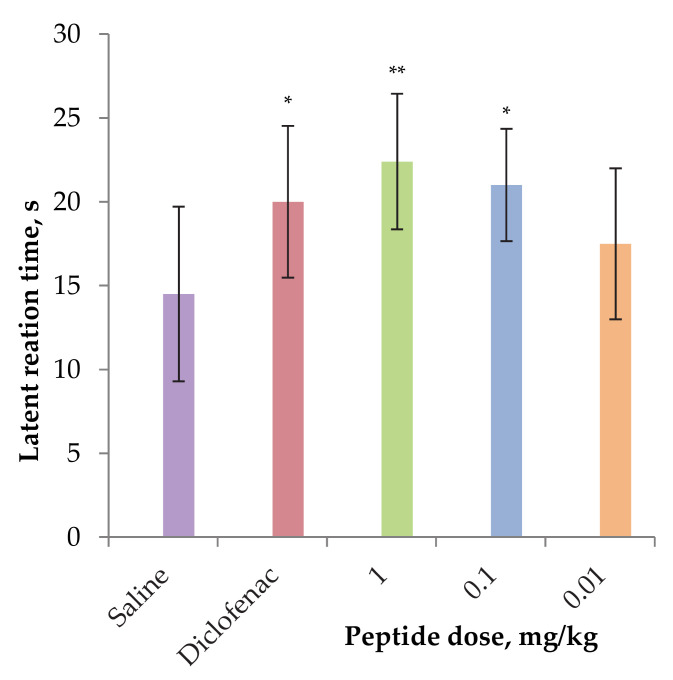
Analgesic effect of AnmTX Sco 9a-1 in the carrageenan-induced thermal hyperalgesia model. Saline buffer as a negative control and Diclofenac at a dose of 1 mg/kg administered intramuscularly as a positive control were used. Results are presented as mean ± SD (n = 7–8). The significance of differences was estimated by the Student’s t-criterion to the saline group. The significance marked as * *p* < 0.05 and ** *p* < 0.01.

The effect of the peptide on acute pain sensitivity in the carrageenan-induced thermal hyperalgesia model was assessed using a hot-plate test after one hour of carrageenan administration. AnmTX Sco 9a-1 demonstrated a significant analgesic effect at doses of 0.1 and 1 mg/kg by reducing the latent time of inflamed paw licking vs. control group (Figure 6). In this model the peptide was more effective than Diclofenac. Summarizing the results above, we concluded that the peptide has both anti-inflammatory and analgesic activities, is not inferior to the currently used NSAIDs like Diclofenac and has prospects for development as drug seeds.

#### 3.3.3. Effect of the AnmTX Sco 9a-1 on Mice Central Nervous System

**Figure 7:**
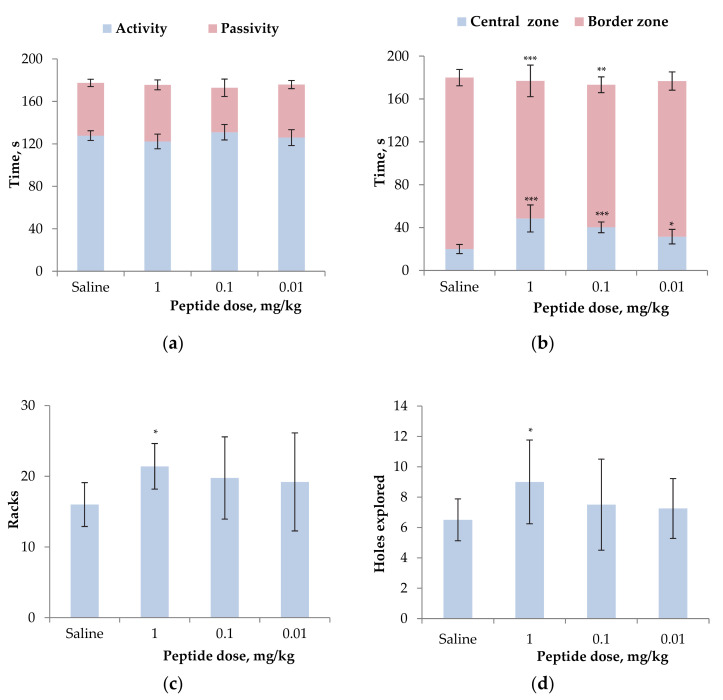
Effect of AnmTX Sco 9a-1 at the doses of 0.01–1 mg/kg on normal mouse behavior in the open field test: time of mouse activity or passivity (**a**), time spent in the central or border zone (**b**), the number of vertical stances (**c**) and peeps into the holes (**d**). Control animals received a similar volume of sterile saline. Results are presented as mean ± SD (n = 7–8). The significance of differences was estimated by the Student’s *t*-criterion to the saline group. Significant differences are presented as * *p* < 0.05, ** *p* < 0.01, and *** *p* < 0.001.

To exclude the possible neurotropic impact to registered anti-inflammatory activity of the peptide, its effect on the motor and orienting-exploratory activity was evaluated in the Open Field test on mice. Three doses identical to those used above were investigated and no depressant effect on CNS was detected (Table S1). Locomotion activity has been slightly lowered for 1 mg/kg dose-treated mice (Figure 7a). Additionally, mice treated with AnmTX Sco 9a-1 at all doses applied spent a longer time in the central zone (about 2 times increase (Figure 7b)), and the time of exit from the central zone increased at 2–3 times as well (Table S1). Two higher doses of the peptide increased the number of vertical stances and peeps into the holes over the control animals significantly (Figure 7c,d). Therefore, the open field test indicated a decrease in the level of anxiety of animals and promotion of animal research behavioral indicators without neurotropic symptoms.

Both anti-inflammation and anti-hyperalgesic effects may be a result of direct action of AnmTX Sco 9a-1 on some nociceptive ion channels. ASICs have been known to participate in inflammation and pain sensation [5], as well as being involved in the formation of a sense of fear and learning [40]. Moreover, ASIC3 channels are one of the molecular targets of Diclofenac [41] that have a similar effect to the peptide, and peptide Pi-actitoxin-Ugr1a shared 41% homology by structure (Figure 3) and also is the inhibitor of ASIC3 [22]. Therefore, the effect of AnmTX Sco 9a-1 was tested on oocytes with a heterologous expression of two main types ASIC1a and ASIC3 channels.

### 3.4. Electrophysiological Effect of AnmTX Sco 9a-1 on ASICs

**Figure 8:**
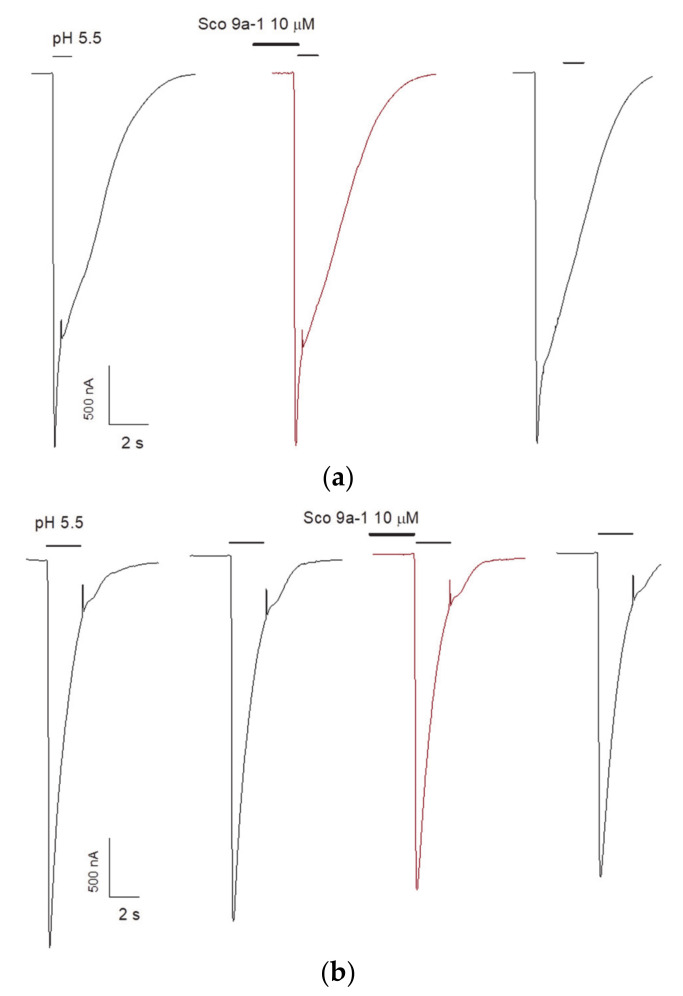
Acid-induced currents through rASIC1a (**a**) and rASIC3 (**b**) expressed in *X. laevis* oocytes were evoked by pH drop from 7.4 to 5.5.

The ability of AnmTX Sco 9a-1 to modulate ASICs was assessed on homomeric rat (r) ASIC1a and ASIC3 channels expressed in *X. laevis* oocytes. An inward current was induced by a rapid pH drop from 7.4 to 5.5, and the peptide was applied 15 s before the acidic pulse. Unfortunately, the peptide demonstrated no effect on rASIC1a or rASIC3 currents (Figure 8). Therefore, its biological activity is probably associated with modulating of another ion channel or receptors of nociception. Considering the limitation of the native peptide amount, the searching for the AnmTX Sco 9a-1 target will be continued after the development of its synthesis or recombinant peptide production technique.

## 4. Conclusions

For the first time, from the swimming sea anemone *S. coccinea* (Actinostolidae family), a biologically active peptide named AnmTX Sco 9a-1 has been isolated and characterized. It is not toxic. It stimulates exploratory motivation and active search behavior, and has an anti-anxiety effect on experimental animals. AnmTX Sco 9a-1 is able to reduce carrageenan-induced inflammation and hyperalgesia in vivo similar to the currently used NSAIDs like Diclofenac. We believe this is not the only anti-inflammatory peptide with β-hairpin fold in this sea anemone. The presence of similar molecular masses in the MS spectra testifies in favor of the existence of a combinatorial library encoding such peptides. Further experiments will be aimed at proving this assumption and search for an AnmTX Sco 9a-1 cellular target.

## Data Availability

Not applicable.

## Supplementary Materials

https://www.mdpi.com/article/10.3390/biom12111705/s1, Figure S1: Identification of the hydroxyproline residue by Edman automated degradation: (a) chromatographic separation of a PTH-amino acid derivatives (standard mixture, Wako Pure Chemicals) with overlapping PTH-L-hydroxyproline analytical standard; (b) chromatographic separation of the PTH-amino acid derivatives generated on the 6th cycle of the AnmTX Sco 9a-1 analysis. A peak corresponding to the PTH-hydroxyproline is marked by lilac vertical stripe; Table S1: Parameters of motor and orienting–exploratory activity of mice treated by AnmTX Sco 9a-1 in Open Field test. Where T (act)—activity time; T (pass)—passivity time; T (c.z.)—time spent on the central zone, s; T (b.z.)—stay on the border zone, s; V, m/s—average travel speed; V(act), m/s—average movement speed during activity; S, m—distance traveled; S (act), m—distance traveled during activity; N (c.z.)—number of visits of central zone; N (s.p.)—number of visits of side platform; N (racks)—vertical activity, the number of racks; N (peeps)—holes explored, the number of peeps into minks; N (def)—number of bowel movements; T (e.c.z.)—time of exit from the central zone, s. Results are presented as mean ± SD (n = 7–8). The significance of differences was estimated by the Student’s t-criterion to the saline group. Significant differences are presented as * *p* < 0.05, ** *p* < 0.01, and *** *p* < 0.001.
