# Supplementary material for: First Anti-Inflammatory Peptide AnmTX Sco 9a-1 from the Swimming Sea Anemone Stomphia coccinea
Source: Biomolecules. 2022 Nov 17;12(11):1705. doi: 10.3390/biom12111705 (PMC9688271; doi:10.3390/biom12111705)
Supplement: Supplementary file 1 [file biomolecules-12-01705-s001.zip › biomolecules-2037447 supplementary.pdf]

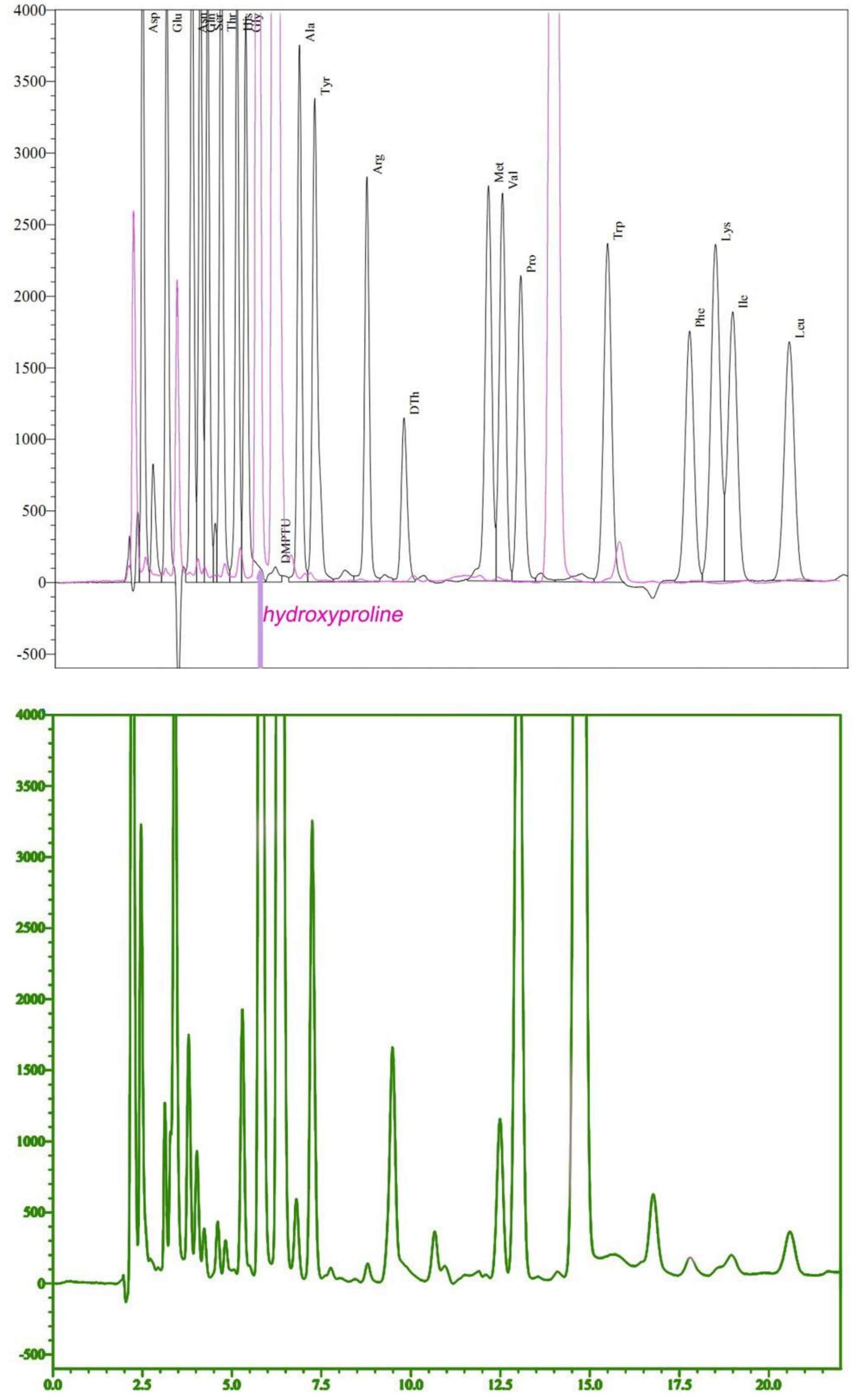

(**a**)

(**b**)

Figure S1: Identification of the hydroxyproline residue by Edman automated degradation: (**a**) chromatographic separation of a PTH-amino acid derivatives (standard mixture, Wako Pure Chemicals) with overlapping PTH-L-hydroxyproline analytical standard; (**b**) chromatographic separation of the PTH-amino acid derivatives generated on the 6th cycle of the AnmTX Sco 9a-1 analysis. A peak corresponding to the PTH-hydroxyproline is marked by lilac vertical stripe.

Table S1. Parameters of motor and orienting–exploratory activity of mice treated by AnmTX Sco 9a-1 in Open Field test.

Group	T (act), s	T (pass), s	T (c.z.), s	T (b.z.), s	V, m/s	V(act), m/s	S, m	S (act), m	N (c.z.)	N (s.p.)	N (racks)	N (peeps)	N (def)	T (e.c.z.), s
**Saline**	127.8±4.6	49.7±3.4	20.1±4.3	160.4±7.6	0.14±0.02	0.19±0.03	24.4±2.9	24.6±2.8	9.3±5.2	9.2±5.6	16.0±3.1	6.5±1.4	1.5±1.8	3.0±1.8
**1 mg/kg**	122.3±7.3	53.3±8.2	48.5±4.9 ***	128.4±7.4 ***	0.15±0.01	0.22±0.02	26.8±2.5	26.8±2.5	16.8±5.2	15.6±5.0	21.4±5.8 *	9.0±3.0 *	1.2±3.8	6.4±3.8
**0.1 mg/kg**	131.0±7.5	41.9±3.8	40.3±6.8 ***	133.2±8.6 **	0.15±0.01	0.21±0.02	26.8±2.7	26.8±2.7	13.8±4.1	14.2±4.6	19.7±6.9	7.5±1.9	2.4±4.8	9.6±4.8
**0.01 mg/kg**	125.9±3.3	49.9±4.9	31.5±5.8 *	145.2±7.2	0.18±0.01	0.25±0.02	28.9±3.4	29.7±1.8	19.6±4.2	19.6±2.9	19.2±1.6	7.25±2.1	1.0±2.7	4.8±2.7

Where T (act)—activity time; T (pass)—passivity time; T (c.z.)—time spent on the central zone, s; T (b.z.)—stay on the border zone, s; V, m/s—average travel speed; V(act), m/s—average movement speed during activity; S, m—distance traveled; S (act), m—distance traveled during activity; N (c.z.)—number of visits of central zone; N (s.p.)—number of visits of side platform; N (racks)—vertical activity, the number of racks; N (peeps)—holes explored, the number of peeps into minks; N (def)—number of bowel movements; T (e.c.z.)—time of exit from the central zone, s. Results are presented as mean ± SD (n = 7–8). The significance of differences was estimated by the Student's t-criterion to the saline group. Significant differences are presented as * $p < 0.05$, ** $p < 0.01$, and *** $p < 0.001$.
